# In-silico selection of peptides for the recognition of imidacloprid

**DOI:** 10.1371/journal.pone.0295619

**Published:** 2023-12-12

**Authors:** Sarah Aldulaijan

**Affiliations:** Chemistry Department, College of Science, Imam Abdulrahman Bin Faisal University, Dammam, Saudi Arabia; The University of Alabama in Huntsville, UNITED STATES

## Abstract

The sensitive detection of pesticides using low-cost receptors designed from peptides can widen their uses in the environmental surveillance for emerging pollutants. In-silico selection of peptides can help accelerate the design of receptor sequence banks for a given target of interest. In this work, we started from *Lymnaea stagnalis* acetylcholine-binding protein Q55R mutant receptor-imidacloprid complex, available in the PDB databank, to select three primary short peptides (YSP09, DMR12, WQW13 respectively having 9, 12 and 13 amino acids (AA) in length) from the pesticide interacting zones with the A, B and C chains of the nicotinic receptor. Using molecular docking and molecular dynamics (MD) simulations, we showed that the three peptides can form complexes with the target imidacloprid, having energies close to that obtained from a reference RNR12 peptide. Combination of these peptides allowed preparing a new set of longer peptides (YSM21, PSM22, PSW31 and WQA34) that have higher stability and affinity as shown by the MM-PBSA calculations. In particular, the WQA34 peptide displayed an average binding free energy of –6.44±0.27 kcal/mol, which is three times higher than that of the reference RNR12 peptide (–2.29±0.25 kcal/mol) and formed a stable complex with imidacloprid. Furthermore, the dissociation constants (K_d_), calculated from the binding free energy, showed that WQA32 (40 μM) has three orders of magnitude lower K_d_ than the reference RNR12 peptide (3.4 × 10^4^ μM). Docking and RMSD scores showed that the WQA34 peptide is potentially selective to the target imidacloprid with respect to acetamiprid and clothianidin. Therefore, this peptide can be used in wet-lab experiments to prepare a biosensor to selectively detect imidacloprid.

## Introduction

Imidacloprid (IMI) is also known as 1-(6-chloro-3-pyridylmethyl)-2-nitroimidazolidine, is a top-selling neonicotinoid insecticide, it functions in the target species as an agonist of nicotinic acetylcholine receptors (nAChRs) [1–7]. It has been widely used for crop protection and animal health due to its excellent efficacy and selectivity to ravaging insects [8]. Based on the following findings from in vitro comparative functional experiments, it has been hypothesized that imidacloprid may be less neurotoxic to mammals, including humans, than to the target insects [2–6, 9]. However, concerns over the safety of mammals have surfaced [1]. According to Yamakuni et al., nAChRs may dysregulate endogenous ACh-elevated catecholamine synthesis in the chromaffin cells of the adrenal gland, ultimately resulting in an increase in adrenaline release from this endocrine organ [1].

Biosensors are currently sought for uses in different fields of applications such diagnostics, food safety and environmental monitoring because of their cost-effectiveness and amiability [10–15]. Peptides have recently been re-examined and their uses as recognition element have been investigated [16–21]. Peptides are known as stable, cost effective and easy to prepare through standard protocols and can be modified to fit specific applications [16, 21–23]. In fact, peptides are suitable for detecting proteins [21, 24], nucleic acids [25], bacteria [26] and chemicals compounds [18, 21, 27]. In addition, peptides can be easily obtained by chemical synthesis methods and avoid the need for in-depth and laborious procedures such as in the case of antibodies [28]. In particular, research activity in peptide-based biosensors is increasing. Based on the search for “peptides” and “biosensor” keywords in SciFinder engine (SciFinder, CAS), several thousands of matches have been found [28].

Detecting the presence of residual levels of pesticides such as imidacloprid, acetamiprid and clothianidin is necessary in many fields such as healthcare, environmental monitoring and food safety [29–33]. Therefore, we examine the binding between IMI compound with several peptides, which were designed by choosing the interacted amino acids in the active site of the Lymnaea stagnalis Acetylcholine-Binding Protein Q55R mutant complex which is very similar structure to insect nicotinic acetylcholine receptors [34]. These peptides will then be guiding models for creating peptide-based biosensors to detect the present of Imidacloprid compound.

## Methods

### Selection of the peptides

In our quest to identify concise peptides suitable for imidacloprid binding, we initiated our investigation using structural insights derived from the *Lymnaea stagnalis* Acetylcholine-Binding Protein Q55R mutant complex (PDB ID 3WTH) [34], accessible within the RCSB PDB database [35]. This pentameric protein represents a pivotal target for neonicotinoid insecticides in the insect kingdom. The architecture of each monomer is intricately composed of a combination of beta sheets and helical structures interconnected by coil regions. The pesticides usually stacked Tyr185 with basic residues such as Lys35 in loop5 [34].

The starting protein is composed of five monomers in complex with five imidacloprid (**S1 Fig**). Three peptides were selected from the binding areas of the different monomer/imidacloprid complexes using the “PLIP” webserver software [36], which enabled us to identify the possible binding sites between the protein and the ligand. The 3D forms of the peptides were retrieved using PEP-FOLD webserver software [37–39].

### Peptides

Based on the results from the selection of peptides and the study of the different poses of interactions between the IMI and the three peptides, we found out that longer peptides have more stable complexes and higher affinity toward the ligand. Therefore, we intentionally provoked several modifications and mutations to create four new peptides having lengths from 21 to 34 residues. Also in this case, we used PEP-FOLD to generate the 3D structures of the peptides. More information about the modifications and mutations are discussed in the Results and Discussion section.

### Molecular docking

To evaluate the affinity between these peptides and imidacloprid, we performed a molecular docking simulation using AutoDock VINA (v1.2.3) [40], with the peptides serving as the receptors. To ensure reproducibility, the entire peptide was positioned within a defined grid box, which had dimensions of 66 × 40 × 40 Å^3^ and the hydrogens were added to the polar atoms of the peptides. Imidacloprid was utilized as the ligand, and it’s important to note that all bonds in the ligand were specified as rotatable, allowing for flexible conformational sampling throughout the docking process. These specific parameters and conditions were employed to substantiate our methods and make them accessible for replication by others. The AutoDock Tools (v1.5.7) [41] was used to prepare the input files. The pH of the systems is 7. PyMOL [42] and BIOVIA Discovery Studio Visualizer [43] were used to analyze the established interactions between the peptides and the ligand.

### MD simulations

To confirm the stability of the selected poses, MD simulations were carried out using NAMD software [44] for a duration of 480 ns. Beforehand, the designed peptide-IMI complexes were minimized and equilibrated using NAMD software for 10,000 cycles. Detailed MD simulations using the complex structures were conducted with the CHARMM36 forcefield [45]. The complexes were solvated in a cubic box with keeping 10 Å between the complex and the box edge. NaCl ions were added to neutralize the system charge. For all simulations, temperature was set at 310 K and the pressure was set to 1 bar to closely mimic the general wet-lab experimental conditions. Subsequently, the fully temperature and pressure equilibrated systems were used as the initial configurations for the MD production. All simulations were conducted using a 2-fs time step.

Root-Mean-Square-Deviation (RMSD) was calculated with tools included in VMD [46] to check the stability of the studied systems. MM–PBSA method [47], implemented in the VMD CaFE plugin [48], was used to calculate the average of free energy from 3 different trajectories between 440 ns and 480 ns simulation time, after the convergence of the systems [47, 49, 50].

The binding free energy (${\Delta G}_{binding}^{slvd}$) can be decomposed into the relative free energy of the solvated receptor–ligand complex (${\Delta G}_{sol}^{Cplx}$) and the separated, solvated ligand (${\Delta G}_{sol}^{Lig}$) and receptor (${\Delta G}_{sol}^{Rec}$) as shown in Eq 1:

$${\Delta G}_{binding}^{slvd}={\Delta G}_{sol}^{Cplx}–{\Delta G}_{sol}^{Rec}–{\Delta G}_{sol}^{Lig}$$ Eq 1

This free energy (${\Delta G}^{s}$) can be further decomposed into four main contributions from the van der Waals (${\Delta E}_{vdW}$), the electrostatic interaction (${\Delta E}_{elec}$), polar solvation (${\Delta G}_{pol}$) and nonpolar solvation (${\Delta G}_{nonpol}$) as shown in Eq 2:

$${\Delta G}^{s}={\Delta E}_{vdW}+{\Delta E}_{elec}+{\Delta G}_{pol}+{\Delta G}_{nonpol}$$ Eq 2

The dissociation constants were calculated from the values of the binding free energy for the different peptide/IMI complexes using the online available applet (URL: https://protsim.github.io/protsim) [51]. The dissociation constant (K_d_) is linked to the free binding energy by the following equation:

$$\Delta G=–RT\;ln\;K_{a}=RT\;ln\;K_{d}$$ Eq 3

Where R is the gas constant, T is the working temperature K_a_ and K_d_ are respectively the association and the dissociation constants. However, based on the information provided in the article [51], we used the method of orthogonal distance regression to calculate the dissociation constant (K_d_) and did not use the theoretical method mentioned in Eq 3. We followed the instructions in the article to input the raw data and leave the calculation method as the default value to obtain the values of K_d_, K_a_, and ΔG. We cited the article to acknowledge the authors and followed the terms of the license for any modifications made to the source code.

## Results and discussion

### Selection of the primary peptides

The analysis of the different complexes between the monomers and imidacloprid showed that the existence of 7 amino acids (143A, 185A, 192A, 53B, 104B, 112B and 114B from the A and B chains) interacting with the target pesticide (S2 Fig). Based on these findings, we selected the sequence from A185 to A192 to build the part I (named: YSP09) and the other from 104B to 114B to build the part II (named: DRM12). A third peptide (named WQW13), consisting of 13 amino acids located between TRP53 and TRP65 from chain C of the protein, was also selected since it interacts with imidacloprid, as confirmed by the PLIP analysis. These results suggest that the interaction between TRP53 and imidacloprid is facilitated by a π-stacking interaction between the imidacloprid molecule and the side chain of tryptophan residue at position 53 in the protein chain. Furthermore, the molecular docking results show that these 3 peptides have good binding affinity scores ranging between –3.17 and –3.80 kcal/mol (entries 1 to 3 from Table 1).

**Table 1:** Sequences of the selected peptides, their codes, number of sequences and binding affinity from docking in kcal/mol.

Entry	Peptide Code ^a^	Sequences	Number of Sequences	Binding Affinity (kcal/mol)
1	**YSP09**	^1^YSCCPEAYP^9^	9	–3.21
2	**DRM12**	^1^DRVVSDGEVLYM^12^	12	–3.17
3	**WQW13**	^1^WQRTTWSDRTLAW^13^	13	–3.80
4	**YSM21**	^1^YSCCPEAYPDRVVSDGEVLYM^21^	21	–4.30
5	**PSM22**	^1^PSHSSDWALTRDSWTTRQWLYM^22^	22	–4.61
6	**PSW31**	^1^PSHSSYSCCPEAYPDRVVSDGEVLYMTTRQW^31^	31	–4.80
7	**WQA34**	^1^WQRTTWYSCCPEAYPDRVVSDGEVLYMSDRTLAW^34^	34	–5.30
8	**RNR12** ^b^	^1^ RNRHTHLRTRPR^12^	12	–3.90

^a^ Peptide code is generated from the first two amino acids from the right followed by the last amino acid and total number of sequences.

^b^ This peptide is studied in literature [27] and is run for sake of comparison.

### Improving the performances of the peptides

**Fig 1:**
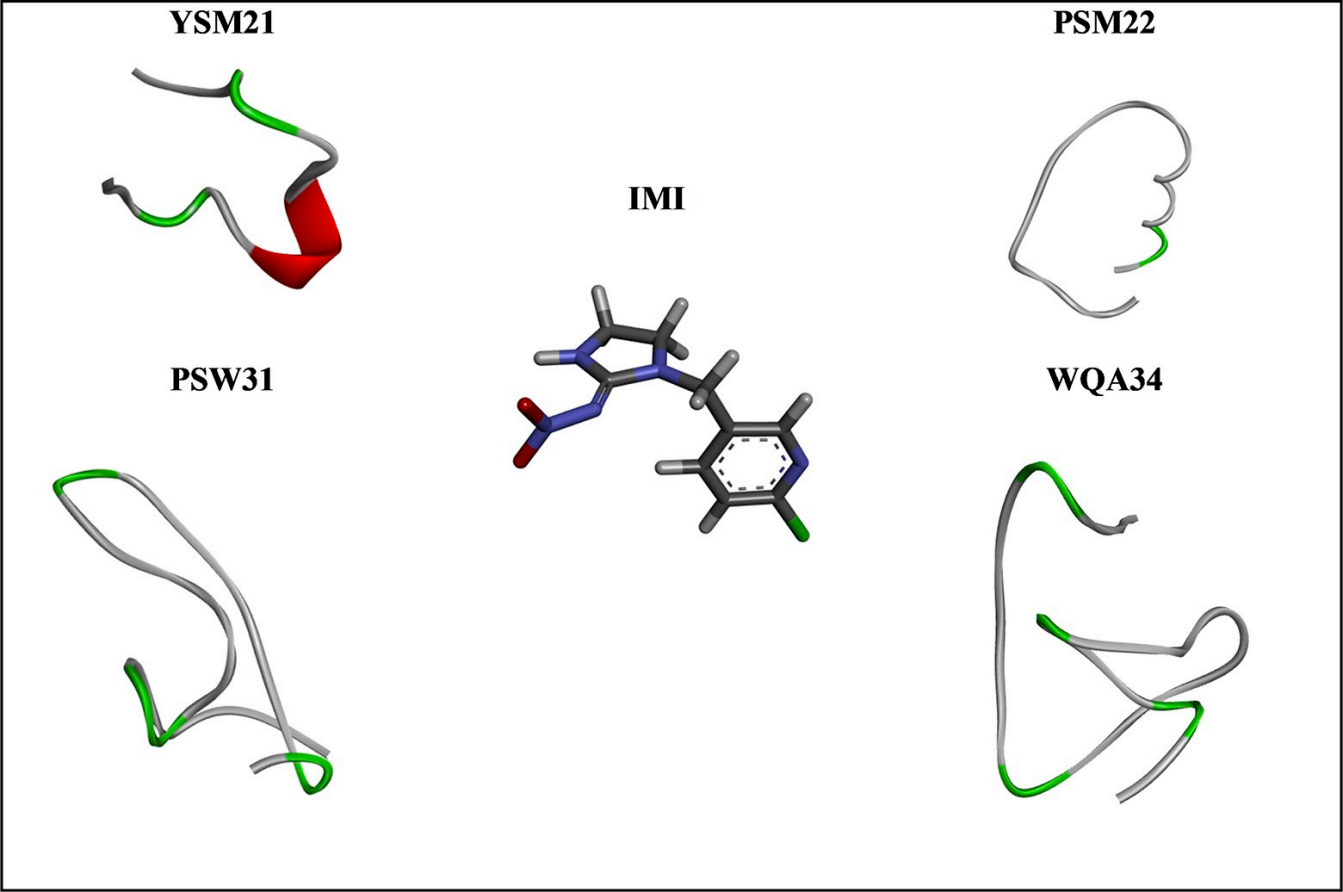
3D structures for IMI and the four peptides YSM21, PSM22, PSW31, and WQA34. In the middle is the structure of the IMI compound surrounded by four peptides YSM21, PSM22, PSW31, and WQA34 which have been obtained from the combination of the initial ones.

To improve the interaction between the peptide sequence and IMI, we combined three shorter peptides (entries 1 to 3 from Table 1) to obtain four longer peptides (entries 4 to 7 from Table 1). YSP09 was linked DRM12 from its N-terminal position to create a 21-amino acid sequence (YSM21), which was tested for interactions with IMI. For (PSM22) peptide, we started from invert order of WQW13 and then we modified it by adding six (PSHSSD) and three (LYM) amino acids in the C-terminal and N-terminal positions, respectively, to build a new peptide with 22-AAs sequence. PSW31 was built from YSM21, and the sequences were modified by adding the first five amino acids of PSM22 (PSHSS) in C-terminal and the first five amino acids of WQW13 in invert order (TTRQW) in the N-terminal position to form a sequence with 31 amino acids. The last peptide (WQA34) is obtained by adding the first six and the last seven amino acids from (WQW13) to C-terminal and N-terminal positions of peptide YSM21, respectively. The 3D structures of the entries 4 to 7 with IMI are shown in Fig 1. After that, the stability, and the affinity of the four combined peptides toward IMI was assessed using molecular docking.

### Molecular docking

**Fig 2:**
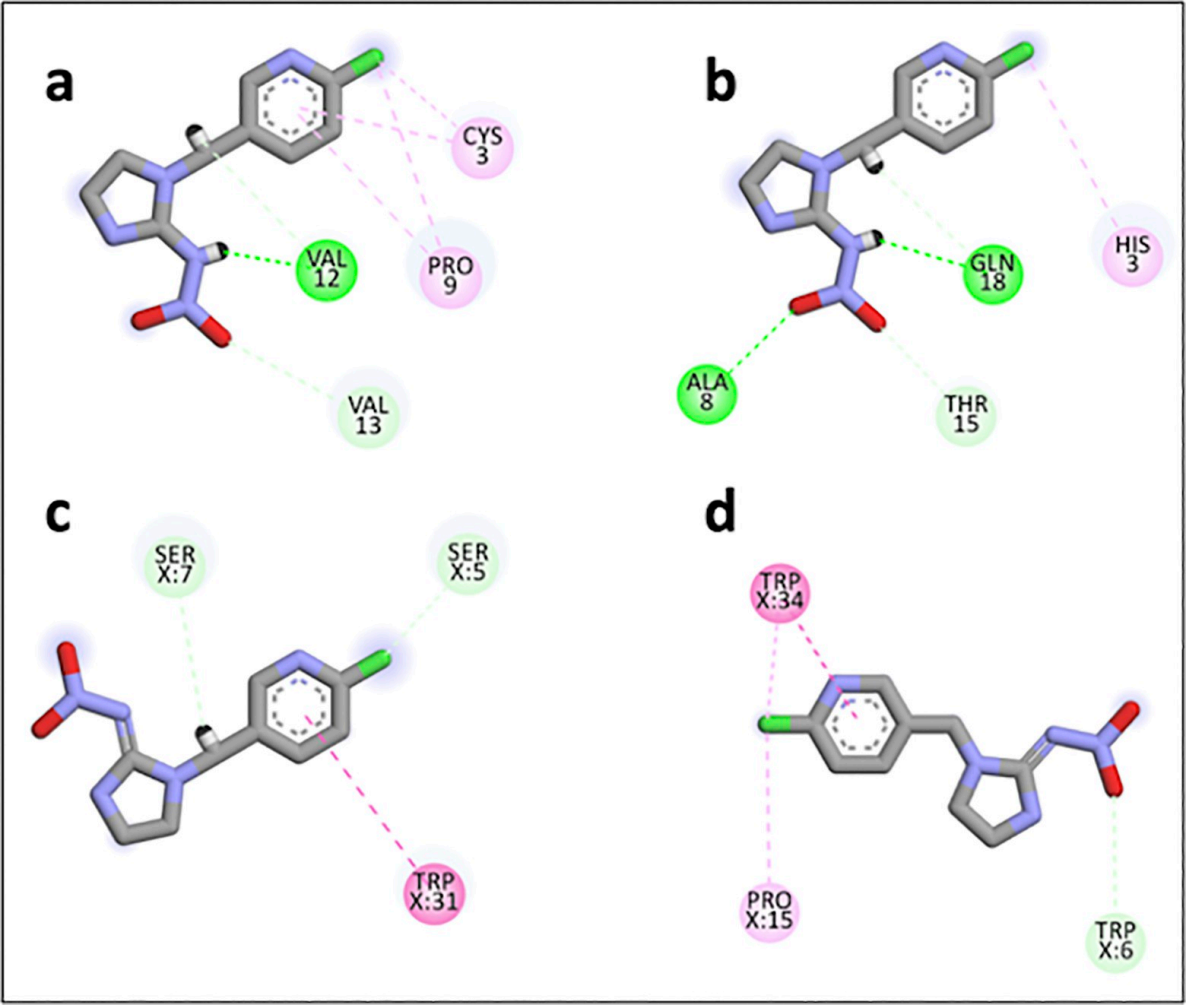
Interactions between IMI and a) YSM21, b) PSM22, c) PSW31 and d) WQA34 form the molecular docking results. The non-covalent interactions established between the IMI compound and the four peptides as the results of the molecular docking generated by BIOVIA Discovery Studio Visualizer software.

From Table 1, the docking results show that the YSM21 have the lowest score docking (–4.30 kcal/mol). This peptide established only 3 hydrogen bonds (HBs) with IMI within less than 4 Å. There is one conventional hydrogen bond from VAL12 (2.33 Å), one carbon HB from VAL12 (2.32 Å), and one carbon HB from VAL13 (3.45 Å). On the other hand, PSM22 has more affinity to the target since it docking score is 35% higher (–4.61 kcal/mol) resulting from several non-covalent interactions with imidacloprid, within 4 Å. We can notice two conventional HBs, one from GLN18 (2.61 Å) and one from ALA8 (4.00 Å) and two carbon HBs from TRP15 (2.40 Å) and from GLN18 (2.38 Å). The binding energy of peptide PSW31 is even higher than that found with PSM22 (–4.80 kcal /mol). The receptor established two carbon short hydrogen interactions (<2.70 Å) with SER5 (2.66 Å) and with SER7 (2.60 Å). WQA34 has the highest score docking among the four peptides under the study (–5.30 kcal/mol), although it shows only one HB with the target from TRP6 (3.91 Å) Fig 2.

From the summarized data in **Table 1**, several preliminary conclusions can be drawn: i) the docking scores of the short peptides are lower than those found for the longer peptides but remain in the same magnitude of RNR12 (–3.80 kcal/mol for WQW13 vs. –3.90 kcal/mol for RNR12), selected by using modified phage display library [27, 52–54], RNR12 is a highly specific oligopeptide sequences for the recognition imidacloprid [27] and ii) the docking scores increased after the lengthening and the combination of the 3 primary selected peptides. Indeed, YSM21 has the lowest score among the four new peptides (–4.30 kcal/mol), but it is higher than the best score found for the short WQW13 and reference RNR12 peptides. Thus, the increasing of sequence length of the peptides increases the binding energy, which is probably due to a decrease of the peptide flexibility, but these conclusions should be confirmed by more accurate MD simulations. The 3D structure of RNR12 and the interactions from molecular docking results are shown in **S3 Fig**, it shows that RNR12 forms two interactions with imidacloprid resulting from the interaction with ARG3 and LEU7.

### MD simulations

#### Thermodynamic analysis

Further work has been done only on the longer peptides (YSM21, PSM22, PSW31 and WQA34) and we compared their results to that obtained with the reference RNR12 peptide. All the data, gathered in Table 2, indicate that the corresponding molecular systems are energetically favorable since they all showed negative free energy values.

**Table 2:** The average free energies result in kcal/mol for the solvated systems, details of the energy contributions for the different complexes and the dissociation constant ^a^.

Complexes	ΔE_Vdw_	ΔE_elec_	ΔG_pol_	ΔG_nonpol_	ΔG_(binding,slvd)_	K_d_ (μM)
	(kcal/mol)	(kcal/mol)	(kcal/mol)	(kcal/mol)	(kcal/mol)	
YSM21/IMI	–2.14	–0.88	0.63	–1.26	–3.54±0.08	1.4 × 10^4^
PSM22/IMI	–1.55	–0.60	0.75	–1.14	–2.64±0.07	1.2 × 10^4^
PSW31/IMI	–1.85	–0.67	1.49	–1.17	–2.43±0.21	3.4 × 10^4^
WQA34/IMI	–5.30	–1.95	2.61	–2.06	–6.44±0.27	40
RNR12/IMI	–1.37	–0.80	1.05	–1.11	–2.29±0.25	3.4 × 10^4^

^a^ The average free energy of solvated receptor-ligand binding (${\Delta G}_{binding,slvd}$) and the four main contributions from the van der Waals (${\Delta E}_{vdW}$), the electrostatic interaction (${\Delta E}_{elec}$), polar solvation (${\Delta G}_{pol}$) and nonpolar solvation (${\Delta G}_{nonpol}$, equilibrium dissociation constant (K_d_).

More in-depth analysis shows that the reference RNR12 peptide performed well in comparison to the results from docking. Indeed, the RNR12 has an average free energy of –2.29±0.25 kcal/mol, which is higher than that obtained with longer PSW31 peptide and slightly lower than that obtained with PSM22. YSM21 and WQA34 resulted in average free energy values higher than that obtained with RNR12. YSM21 and WQA34 have average free energy values higher by 154.6 ±3.2% and 281.2 ±1.1% in respect to the value obtained for RNR12. Potentially these two peptides can serve for a better recognition of the target molecule than the reference peptide.

Furthermore, energy decomposition showed that the main contribution to the free energy arises from van der Waals and the electrostatic interactions, and nonpolar free energy. The polar free energy contributed unfavorably to the interaction since all the values are positive but remain weak compared to the other contributions. For instance, the favorable contribution is 144.6% of the interaction energy while the unfavorable contribution is 40.5%. The small difference comes from two weak terms which are a favorable entropic one and unfavorable dispersion free energy term, neglected in Eq 2.

From the data gathered in **Table 2**, we can see that the YSM21, PSM22, PSW31 peptides have high dissociation constants (1.4 × 10^4^ to 3.4 × 10^4^ μM), which are in the same order of magnitude of the reference peptide. This denotes that these complexes are not very stable because their relatively low average free binding energies. Peptide WQA34 has a dissociation constant of 40 μM, which is 3 orders of magnitude lower than the reference RNR12 peptide, making this in-silico designed peptide a viable candidate for the recognition of imidacloprid and building sensors for it.

#### RMSD analysis

**Fig 3:**
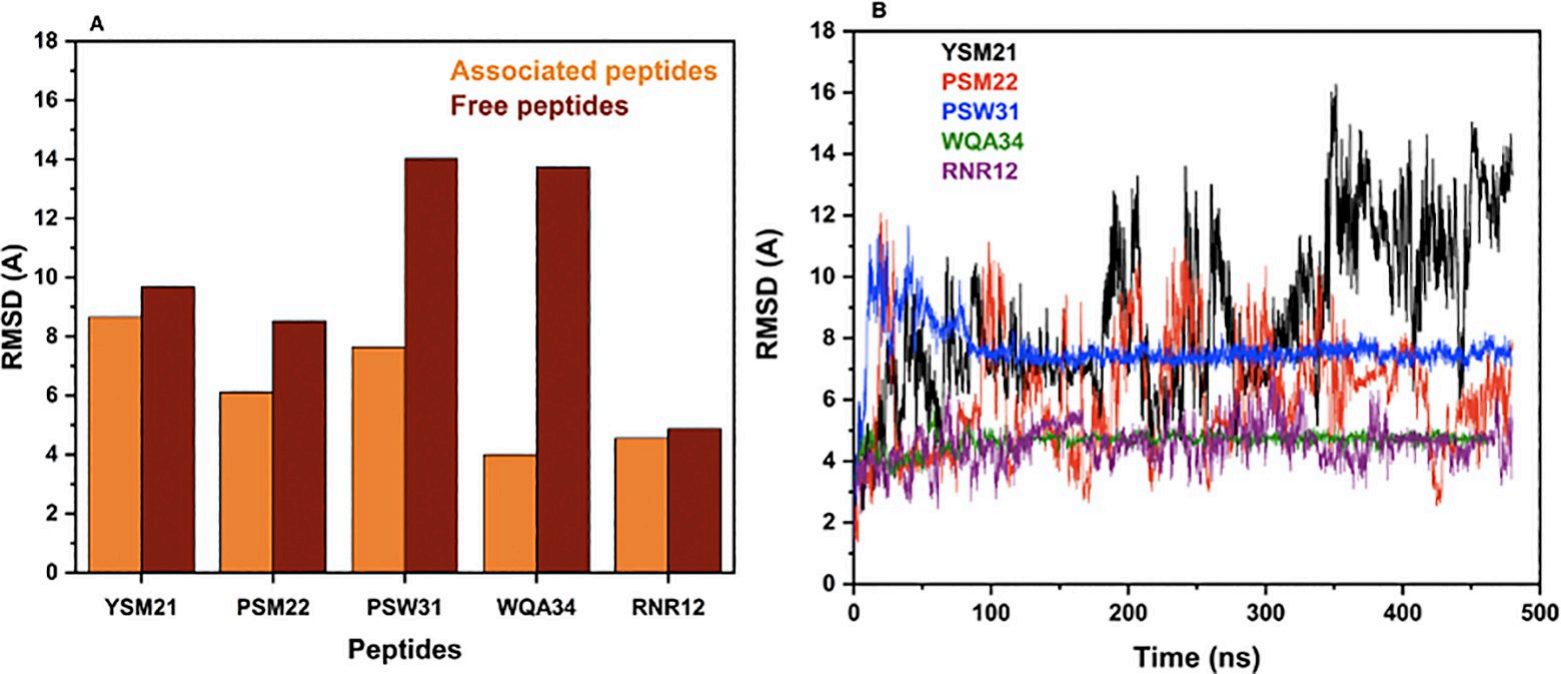
The molecular dynamic results: A) the root mean square deviation (RMSD) for the free and the associated peptides, B) The root mean square deviation (RMSD) for the peptides vs time. (The root mean square deviation (RMSD) in Å for the five peptides YSM21, PSM22, PSW31, WQA34 and RNR1: the free peptides (in red color) and the associated peptides (in orange color); B) The root mean square deviation (RMSD) for these five peptides vs time in nano second).

The RMSD analysis indicates that there are significant changes in the IMI-peptide interactions between the peptide-free and associated states. Specifically, the RMSD values of PSW31 and WQA34 show a substantial decrease of 50% and 74%, respectively, indicating a more stable interaction with the peptide in the associated state Fig 3A. The reference RNR12 peptide displayed a RMSD almost superposable to that of WQA34, showing that both peptides are stable. On the other hand, YSM21 exhibits only minor variations in RMSD that do not exceed 10%, suggesting that the peptide-IMI interaction is relatively stable. From Fig 3B, we can see that a large fluctuation of the RMSD occurred denoting the ligand keeps changing confirmation during the overall simulation time. PSM22 displayed a significant decrease in the average RMSD value of 30% in the associated state compared to the free state, indicating that the complex formation has a stabilizing effect on the peptide conformation.

Overall, these results provide valuable insights into the IMI-peptide interactions and their dynamics, which can be useful in understanding the underlying mechanisms of biological processes. The time-dependent RMSD analysis reveals that the peptides PSW31 and WQA34 reach stability after 100 ns of simulation, while YSM21 and PSM22 showed RMSD fluctuations till the end of the simulation time, although they are a large number of contacts (09) with the target, as shown in S4 Fig. This difference in stability can be attributed to the length of the peptide sequences, which increased from 21 to 34 in the case of PSW31 and WQA34. The longer peptide sequences may provide more stabilizing interactions with the pesticide molecule and reduce the fluctuation in the IMI-peptide complex structure, leading to a faster stabilization. Conversely, the shorter peptide sequences of YSM21 and PSM22 may not provide enough stabilizing interactions, resulting in higher structural fluctuations and slower stabilization. These findings highlight the importance of considering the length and sequence of peptides in studying their interactions with proteins and the dynamic behavior of imidacloprid-peptide complexes over time.

### Selectivity

**Fig 4:**
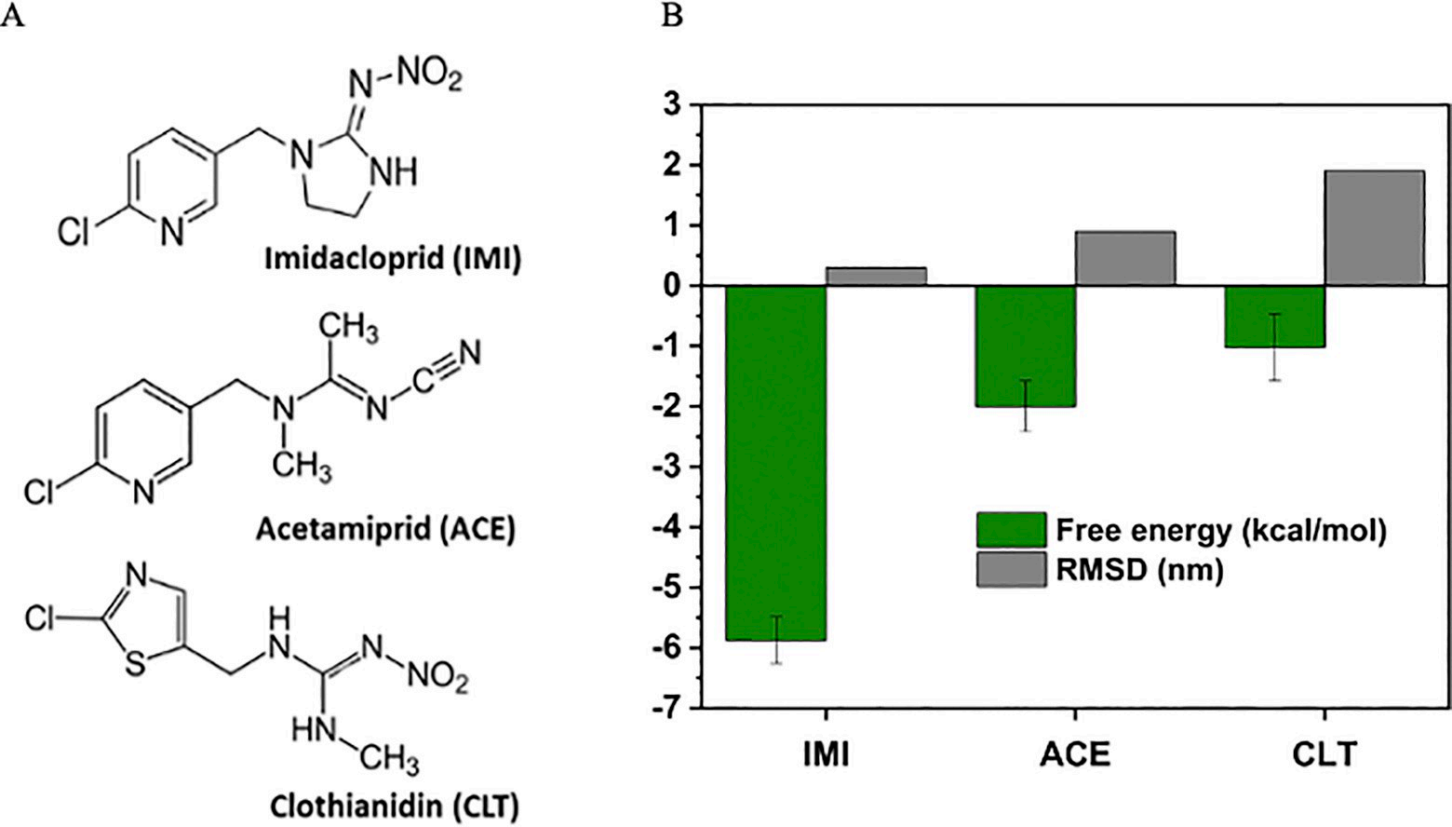
A) Chemical structure of the three neonicotinoid pesticides and B) Plots of root mean square deviation (RMSD) and the average free energy determined for the selectivity of WQA34 peptide (the error bars are obtained from three different trajectories). Figure (A) shows the 2D chemical structure of the three neonicotinoid pesticides IMI: imidacloprid, ACE: acetamiprid, CLT: Clothianidin and Figure (B) shows the plots of root mean square deviation (RMSD) in Å (grey color) and MM-PBSA results in kcal/mol with the error bars (green color) determined for the selectivity of WQA34 peptide.

We also examined the selectivity of the lead peptide (i.e. WQA34) by comparing it with two other pesticides. The first one is acetamiprid (ACE) [30] and the second one is clothianidin (CLT) [7, 9], which are two widely used neonicotinoid insecticides from the same family **Fig 4A** [9, 30]. As shown in **Fig 4B**, The MM-PBSA results of IMI, ACE and CLT were respectively –5.88±0.38 kcal/mol, –2.00±0.48 kcal/mol and –1.02±0.55 kcal/mol, demonstrating a higher affinity of WQA34 to the target pesticide although there is structural similarity between them. This is confirmed by the RMSD values (IMI: 0.3, ACE: 0.9, CLT: 1.9). Indeed, we witnessed a higher stabilization of the complex formed with IMI in respect to those formed with ACE or CLT. The RSMD score of CLT is approximately six times higher than that found for IMI and its docking score is lower by 66%. This suggests that the selected peptide is potentially selective to IMI in the following order IMI>ACE>CLT.

## Conclusion

Starting from nicotinic receptor-imidacloprid complex available in the PDB databank, we selected three primary short peptides that can complex the target molecule with energies close to that obtained from a reference RNR12 peptide, selected in wet-lab experiment using Phage display. The combination of these peptides allowed their lengthening and increasing of the overall stability. The latter showed higher affinity to the target as showed by the MM-PBSA method. In particular, the WQA34 peptide has a threefold higher affinity and 850 times lower dissociation constant with the target compared to the reference peptide and showed that it formed a stable complex with imidacloprid as demonstrated by MD simulations. Furthermore, the peptide showed to be selective to the target pesticide against two other members from the same family. The WQA34 can be used in wet-lab experiments to selectively recognize the target pesticide. Still these data showed be taken with care because of the inherent approximations used in the MD simulations and the effect of pH which could influence considerably the calculated values of binding free energy and the dissociation constants.

Commencing with the nicotinic receptor-imidacloprid complex found in the PDB database, three initial short peptides were identified capable of forming complexes with the target molecule, exhibiting energies closely aligned with those of a reference RNR12 peptide, which was selected through wet-lab experiments using Phage display. Combining the peptides led to the lengthening of the peptide sequences and enhancement in overall stability. In particular, the WQA34 peptide exhibited a threefold increase in affinity and an 850-fold reduction in dissociation constant when compared to the reference peptide. Molecular dynamics simulations provided evidences that WQA34 formed a robust and stable complex with imidacloprid. Moreover, these investigations revealed the peptide’s selectivity towards the target pesticide when compared to two other members of the same chemical family. Overall, these results indicate that WQA34 holds significant potential for application in wet-lab experiments, enabling the selective recognition of the target pesticide. However, it is essential to exercise caution when interpreting these findings, considering the inherent approximations associated with MD simulations and the potential influence of pH on the calculated values of binding free energy and dissociation constants.

## Supporting information

S1 Fig The structural of the *Lymnaea stagnalis* Acetylcholine-Binding Protein Q55R mutant complex (PDB ID 3WTH). (DOCX)

S2 Fig The 3D structures of the (A) position of IMI between chain A and chain B (B) interactions between IMI and the chain A and B residues.
(DOCX)

S3 Fig A) The 3D-structure of RNR12 peptide B) The interactions between IMI and RNR12 ligand from molecular docking calculations.
(DOCX)

S4 Fig A) The number of contacts for the peptides vs time B) The minimum number of contacts for each peptide.
(DOCX)

## References

[1] Kawahata I, Yamakuni T. Imidacloprid, a neonicotinoid insecticide, facilitates tyrosine hydroxylase transcription and phenylethanolamine N-methyltransferase mRNA expression to enhance catecholamine synthesis and its nicotine-evoked elevation in PC12D cells. Toxicology. 2018; 394:84–92. Epub 20171212. doi: 10.1016/j.tox.2017.12.004 . 29246838

[2] Ihara M, Matsuda K, Otake M, Kuwamura M, Shimomura M, Komai K, et al. Diverse actions of neonicotinoids on chicken α7, α4β2 and Drosophila–chicken SADβ2 and ALSβ2 hybrid nicotinic acetylcholine receptors expressed in Xenopus laevis oocytes. Neuropharmacology. 2003;45(1):133–44. doi: 10.1016/S0028-3908(03)00134-5 12814666

[3] Matsuda K, Buckingham SD, Freeman JC, Squire MD, Baylis HA, Sattelle DB. Effects of the alpha subunit on imidacloprid sensitivity of recombinant nicotinic acetylcholine receptors. Br J Pharmacol. 1998;123(3):518–24. doi: 10.1038/sj.bjp.0701618 ; PubMed Central PMCID: PMC1565179. 9504393 PMC1565179

[4] Tomizawa M, Casida JE. Minor structural changes in nicotinoid insecticides confer differential subtype selectivity for mammalian nicotinic acetylcholine receptors. Br J Pharmacol. 1999;127(1):115–22. doi: 10.1038/sj.bjp.0702526 ; PubMed Central PMCID: PMC1566001. 10369463 PMC1566001

[5] Tomizawa M, Casida JE. Neonicotinoid insecticide toxicology: mechanisms of selective action. Annu Rev Pharmacol Toxicol. 2005; 45:247–68. doi: 10.1146/annurev.pharmtox.45.120403.095930 . 15822177

[6] Tomizawa M, Casida JE. Selective toxicity of neonicotinoids attributable to specificity of insect and mammalian nicotinic receptors. Annu Rev Entomol. 2003; 48:339–64. Epub 20020604. doi: 10.1146/annurev.ento.48.091801.112731 . 12208819

[7] Brown LA, Ihara M, Buckingham SD, Matsuda K, Sattelle DB. Neonicotinoid insecticides display partial and super agonist actions on native insect nicotinic acetylcholine receptors. J Neurochem. 2006;99(2):608–15. Epub 20060808. doi: 10.1111/j.1471-4159.2006.04084.x . 16899070

[8] Tian J, Zhang Q, An X, Liu H, Liu Y. Molecular Dynamics Simulations Study on the Resistant Mechanism of Insects to Imidacloprid due to Y151-S and R81T Mutations in nAChRs. Mol Inform. 2019;38(8–9):e1800125. Epub 20190711. doi: 10.1002/minf.201800125 . 31294911

[9] Dai P, Jack CJ, Mortensen AN, Bustamante TA, Bloomquist JR, Ellis JD. Chronic toxicity of clothianidin, imidacloprid, chlorpyrifos, and dimethoate to Apis mellifera L. larvae reared in vitro. Pest Manag Sci. 2019;75(1):29–36. Epub 20180904. doi: 10.1002/ps.5124 . 29931787

[10] Baachaoui S, Mabrouk W, Charradi K, Slimi B, Ramadan AM, Elsamra RMI, et al. Laser-induced porous graphene electrodes from polyketimine membranes for paracetamol sensing. Royal Society Open Science. 2023; 10:230294. doi: 10.1098/rsos.230294 37538749 PMC10394415

[11] Meftah M, Habel A, Baachaoui S, Yaacoubi-Loueslati B, Raouafi N. Sensitive electrochemical detection of polymorphisms in IL6 and TGF beta 1 genes from ovarian cancer DNA patients using EcoRI and DNA hairpin-modified gold electrodes. Microchim Acta. 2023;190(1) 15. doi: 10.1007/s00604-022-05595-w WOS:000895688500002. 36479645

[12] Baachaoui S, Mastouri M, Meftah M, Yaacoubi-Loueslati B, Raouafi N. A Magnetoelectrochemical Bioassay for Highly Sensitive Sensing of Point Mutations in Interleukin-6 Gene Using TMB as a Hybridization Intercalation Indicator. Biosensors-Basel. 2023;13(2). doi: 10.3390/bios13020240 WOS:000938893100001. 36832006 PMC9954083

[13] Ouedraogo B, Baachaoui S, Tall A, Tapsoba I, Raouafi N. Laser-induced graphene electrodes on polyimide membranes modified with gold nanoparticles for the simultaneous detection of dopamine and uric acid in human serum. Microchim Acta. 2023;190(8):316. doi: 10.1007/s00604-023-05909-6 37480385

[14] Algethami FK, Rabti A, Mastouri M, Abdulkhair BY, Ben Aoun S, Raouafi N. Highly sensitive capacitance-based nitrite sensing using polydopamine/AuNPs-modified screen-printed carbon electrode. Rsc Adv. 2023;13(31):21336–44. doi: 10.1039/d3ra03898j WOS:001026433500001. 37465569 PMC10350640

[15] Algethami FK, Rabti A, Mastouri M, Ben Aoun S, Abdulkhair BY, Raouafi N. In silico selection of an aptamer for the design of aptamer-modified magnetic beads bearing ferrocene co-immobilized label for capacitive detection of acetamiprid. Talanta. 2023;258. doi: 10.1016/j.talanta.2023.124445 WOS:000951964100001. 36924636

[16] Mastouri M, Baachaoui S, Mosbah A, Raouafi N. In silico screening for oligopeptides useful as capture and reporting probes for interleukin-6 biosensing. RSC Adv. 2022;12(21):13003–13. Epub 20220428. doi: 10.1039/d2ra01496c ; PubMed Central PMCID: PMC9049833. 35497015 PMC9049833

[17] Pavan S, Berti F. Short peptides as biosensor transducers. Anal Bioanal Chem. 2012;402(10):3055–70. Epub 20111215. doi: 10.1007/s00216-011-5589-8 . 22169951

[18] Barbosa AJM, Oliveira AR, Roque ACA. Protein- and Peptide-Based Biosensors in Artificial Olfaction. Trends Biotechnol. 2018;36(12):1244–58. Epub 20180910. doi: 10.1016/j.tibtech.2018.07.004 ; PubMed Central PMCID: PMC6245566. 30213453 PMC6245566

[19] Puiu M, Bala C. Peptide-based biosensors: From self-assembled interfaces to molecular probes in electrochemical assays. Bioelectrochemistry. 2018;120:66–75. Epub 20171123. doi: 10.1016/j.bioelechem.2017.11.009 . 29182910

[20] Liu Q, Wang J, Boyd BJ. Peptide-based biosensors. Talanta. 2015;136:114–27. Epub 20150108. doi: 10.1016/j.talanta.2014.12.020 . 25702993

[21] Erak M, Bellmann-Sickert K, Els-Heindl S, Beck-Sickinger AG. Peptide chemistry toolbox—Transforming natural peptides into peptide therapeutics. Bioorg Med Chem. 2018;26(10):2759–65. Epub 20180131. doi: 10.1016/j.bmc.2018.01.012 . 29395804

[22] Zambrano-Mila MS, Blacio KES, Vispo NS. Peptide Phage Display: Molecular Principles and Biomedical Applications. Ther Innov Regul Sci. 2020;54(2):308–17. Epub 20200106. doi: 10.1007/s43441-019-00059-5 ; PubMed Central PMCID: PMC7222141. 32072579 PMC7222141

[23] MacCulloch T, Buchberger A, Stephanopoulos N. Emerging applications of peptide-oligonucleotide conjugates: bioactive scaffolds, self-assembling systems, and hybrid nanomaterials. Org Biomol Chem. 2019;17(7):1668–82. doi: 10.1039/c8ob02436g . 30483688

[24] Arya SK, Kongsuphol P, Wong CC, Polla LJ, Park MK. Label free biosensor for sensitive human influenza virus hemagglutinin specific antibody detection using coiled-coil peptide modified microelectrode array based platform. Sensors and Actuators B: Chemical. 2014;194:127–33. doi: 10.1016/j.snb.2013.12.066

[25] O’Neil KT, Hoess RH, DeGrado WF. Design of DNA-binding peptides based on the leucine zipper motif. Science. 1990;249(4970):774–8. doi: 10.1126/science.2389143 . 2389143

[26] Pardoux É, Boturyn D, Roupioz Y. Antimicrobial Peptides as Probes in Biosensors Detecting Whole Bacteria: A Review. Molecules. 2020;25(8). Epub 20200424. doi: 10.3390/molecules25081998 ; PubMed Central PMCID: PMC7221689. 32344585 PMC7221689

[27] Ding X, Zhang W, Cheng D, He J, Yang KL. Oligopeptides functionalized surface plasmon resonance biosensors for detecting thiacloprid and imidacloprid. Biosens Bioelectron. 2012;35(1):271–6. Epub 20120307. doi: 10.1016/j.bios.2012.02.060 . 22459587

[28] Sfragano PS, Moro G, Polo F, Palchetti I. The Role of Peptides in the Design of Electrochemical Biosensors for Clinical Diagnostics. Biosensors (Basel). 2021;11(8). Epub 20210723. doi: 10.3390/bios11080246 ; PubMed Central PMCID: PMC8391273. 34436048 PMC8391273

[29] Tsagkaris AS, Pulkrabova J, Hajslova J. Optical Screening Methods for Pesticide Residue Detection in Food Matrices: Advances and Emerging Analytical Trends. Foods. 2021;10(1). Epub 20210105. doi: 10.3390/foods10010088 ; PubMed Central PMCID: PMC7824741. 33466242 PMC7824741

[30] Hamami M, Raouafi N, Korri-Youssoufi H. Self-Assembled MoS2/ssDNA Nanostructures for the Capacitive Aptasensing of Acetamiprid Insecticide. Applied Sciences [Internet]. 2021; 11(4):1382.

[31] Xu Y, Zhang W, Shi J, Li Z, Huang X, Zou X, et al. Impedimetric aptasensor based on highly porous gold for sensitive detection of acetamiprid in fruits and vegetables. Food Chemistry. 2020;322:126762. doi: 10.1016/j.foodchem.2020.126762 32283369

[32] Creedon N, Lovera P, Moreno JG, Nolan M, O’Riordan A. Highly Sensitive SERS Detection of Neonicotinoid Pesticides. Complete Raman Spectral Assignment of Clothianidin and Imidacloprid. J Phys Chem A. 2020;124(36):7238–47. Epub 20200828. doi: 10.1021/acs.jpca.0c02832 . 32701286

[33] El-Akaad S, Mohamed MA, Abdelwahab NS, Abdelaleem EA, De Saeger S, Beloglazova N. Capacitive sensor based on molecularly imprinted polymers for detection of the insecticide imidacloprid in water. Sci Rep. 2020;10(1):14479. Epub 20200902. doi: 10.1038/s41598-020-71325-y ; PubMed Central PMCID: PMC7468110. 32879399 PMC7468110

[34] Ihara M, Okajima T, Yamashita A, Oda T, Asano T, Matsui M, et al. Studies on an acetylcholine binding protein identify a basic residue in loop G on the β1 strand as a new structural determinant of neonicotinoid actions. Mol Pharmacol. 2014;86(6):736–46. Epub 20140929. doi: 10.1124/mol.114.094698 . 25267717

[35] Berman HM, Westbrook J, Feng Z, Gilliland G, Bhat TN, Weissig H, et al. The Protein Data Bank. Nucleic Acids Res. 2000;28(1):235–42. doi: 10.1093/nar/28.1.235 ; PubMed Central PMCID: PMC102472. 10592235 PMC102472

[36] Salentin S, Schreiber S, Haupt VJ, Adasme MF, Schroeder M. PLIP: fully automated protein-ligand interaction profiler. Nucleic Acids Res. 2015;43(W1):W443–7. Epub 2015/04/14. doi: 10.1093/nar/gkv315 ; PubMed Central PMCID: PMC4489249. 25873628 PMC4489249

[37] Thévenet P, Shen Y, Maupetit J, Guyon F, Derreumaux P, Tufféry P. PEP-FOLD: an updated de novo structure prediction server for both linear and disulfide bonded cyclic peptides. Nucleic Acids Res. 2012;40(Web Server issue):W288–93. Epub 20120511. doi: 10.1093/nar/gks419 ; PubMed Central PMCID: PMC3394260. 22581768 PMC3394260

[38] Lamiable A, Thévenet P, Rey J, Vavrusa M, Derreumaux P, Tufféry P. PEP-FOLD3: faster de novo structure prediction for linear peptides in solution and in complex. Nucleic Acids Res. 2016;44(W1):W449–54. Epub 20160429. doi: 10.1093/nar/gkw329 ; PubMed Central PMCID: PMC4987898. 27131374 PMC4987898

[39] Shen Y, Maupetit J, Derreumaux P, Tuffery P. Improved PEP-FOLD Approach for Peptide and Miniprotein Structure Prediction. J Chem Theory Comput. 2014;10(10):4745–58. doi: 10.1021/ct500592m . 26588162

[40] Trott O, Olson AJ. AutoDock Vina: improving the speed and accuracy of docking with a new scoring function, efficient optimization, and multithreading. J Comput Chem. 2010;31(2):455–61. Epub 2009/06/06. doi: 10.1002/jcc.21334 ; PubMed Central PMCID: PMC3041641. 19499576 PMC3041641

[41] Morris GM, Huey R, Lindstrom W, Sanner MF, Belew RK, Goodsell DS, et al. AutoDock4 and AutoDockTools4: Automated docking with selective receptor flexibility. J Comput Chem. 2009;30(16):2785–91. doi: 10.1002/jcc.21256 ; PubMed Central PMCID: PMC2760638. 19399780 PMC2760638

[42] The PyMOL Molecular Graphics System. NY, USA: Schrodinger, LLC. 2010.

[43] BIOVIA DS. BIOVIA Discovery Studio Visualizer, v16.1.0.15350. San Diego: Dassault Systemes; 2015.

[44] Phillips JC, Hardy DJ, Maia JDC, Stone JE, Ribeiro JV, Bernardi RC, et al. Scalable molecular dynamics on CPU and GPU architectures with NAMD. J Chem Phys. 2020;153(4):044130. doi: 10.1063/5.0014475 ; PubMed Central PMCID: PMC7395834. 32752662 PMC7395834

[45] Vanommeslaeghe K, Hatcher E, Acharya C, Kundu S, Zhong S, Shim J, et al. CHARMM general force field: A force field for drug-like molecules compatible with the CHARMM all-atom additive biological force fields. J Comput Chem. 2010;31(4):671–90. doi: 10.1002/jcc.21367 ; PubMed Central PMCID: PMC2888302. 19575467 PMC2888302

[46] Humphrey W, Dalke A, Schulten K. VMD: visual molecular dynamics. J Mol Graph. 1996;14(1):33–8, 27–8. Epub 1996/02/01. doi: 10.1016/0263-7855(96)00018-5 . 8744570

[47] Genheden S, Ryde U. The MM/PBSA and MM/GBSA methods to estimate ligand-binding affinities. Expert Opin Drug Discov. 2015;10(5):449–61. Epub 20150402. doi: 10.1517/17460441.2015.1032936 ; PubMed Central PMCID: PMC4487606. 25835573 PMC4487606

[48] Liu H, Hou T. CaFE: a tool for binding affinity prediction using end-point free energy methods. Bioinformatics. 2016;32(14):2216–8. Epub 2016/05/07. doi: 10.1093/bioinformatics/btw215 . 27153651

[49] Wang E, Sun H, Wang J, Wang Z, Liu H, Zhang JZH, et al. End-Point Binding Free Energy Calculation with MM/PBSA and MM/GBSA: Strategies and Applications in Drug Design. Chemical Reviews. 2019;119(16):9478–508. doi: 10.1021/acs.chemrev.9b00055 31244000

[50] Genheden S, Ryde U. How to obtain statistically converged MM/GBSA results. J Comput Chem. 2010;31(4):837–46. doi: 10.1002/jcc.21366 . 19598265

[51] Pääkkönen J, Jänis J, Rouvinen J. Calculation and Visualization of Binding Equilibria in Protein Studies. ACS Omega. 2022;7(12):10789–95. Epub 20220316. doi: 10.1021/acsomega.2c00560 ; PubMed Central PMCID: PMC8973030. 35382263 PMC8973030

[52] Devlin JJ, Panganiban LC, Devlin PE. Random peptide libraries: a source of specific protein binding molecules. Science. 1990;249(4967):404–6. doi: 10.1126/science.2143033 . 2143033

[53] Jaworski JW, Raorane D, Huh JH, Majumdar A, Lee SW. Evolutionary screening of biomimetic coatings for selective detection of explosives. Langmuir. 2008;24(9):4938–43. Epub 20080326. doi: 10.1021/la7035289 . 18363413

[54] Scott JK, Smith GP. Searching for peptide ligands with an epitope library. Science. 1990;249(4967):386–90. doi: 10.1126/science.1696028 . 1696028

