# Supplementary material for: In-silico selection of peptides for the recognition of imidacloprid
Source: PLoS One. 2023 Dec 12;18(12):e0295619. doi: 10.1371/journal.pone.0295619 (PMC10715655; doi:10.1371/journal.pone.0295619)
Supplement: S1 Fig — (DOCX) [file pone.0295619.s001.docx]

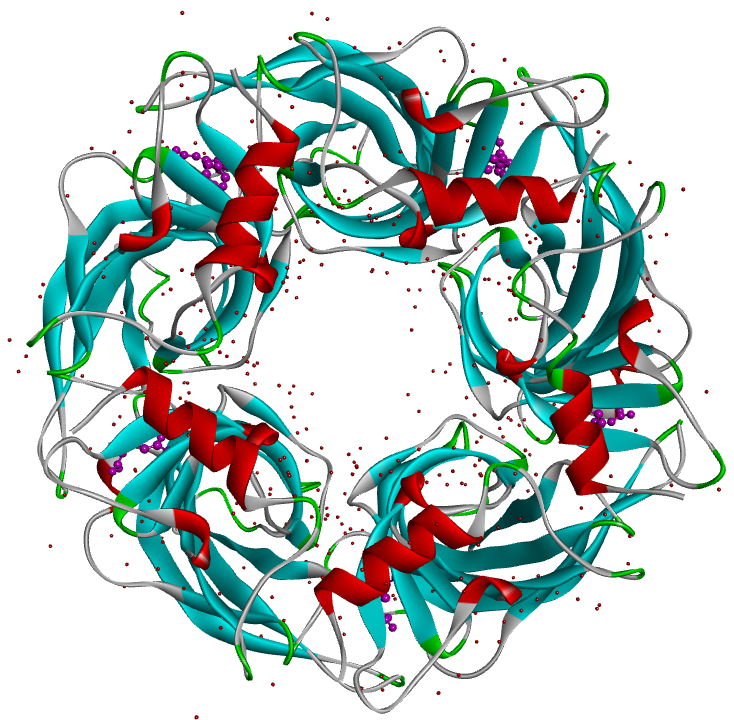


**S1 Fig.** The structural of the Lymnaea stagnalis Acetylcholine-Binding Protein Q55R mutant complex (PDB ID 3WTH)
