# Supplementary material for: In-silico selection of peptides for the recognition of imidacloprid
Source: PLoS One. 2023 Dec 12;18(12):e0295619. doi: 10.1371/journal.pone.0295619 (PMC10715655; doi:10.1371/journal.pone.0295619)
Supplement: S2 Fig — The 3D structures of the (A) position of IMI between chain A and chain B (B) interactions between IMI and the chain A and B residues. (DOCX) [file pone.0295619.s002.docx]

| **A**  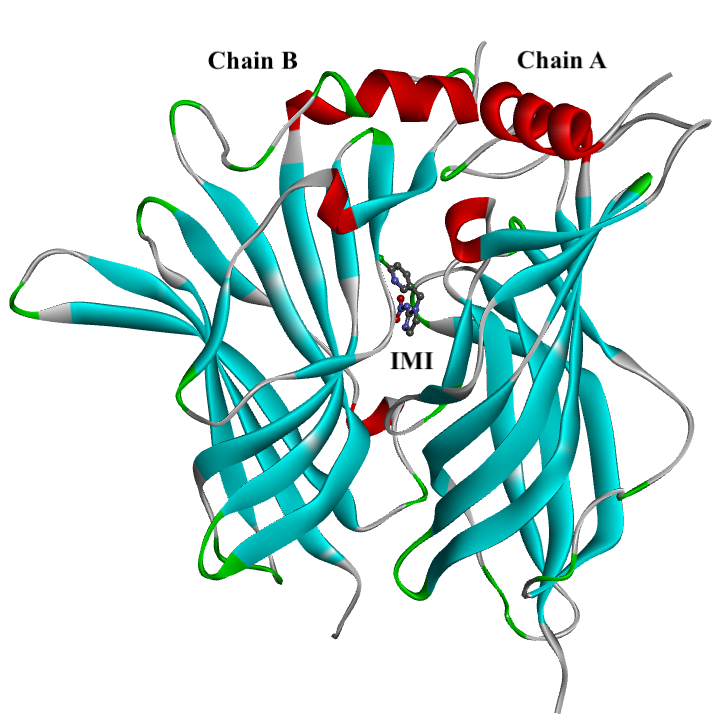 | 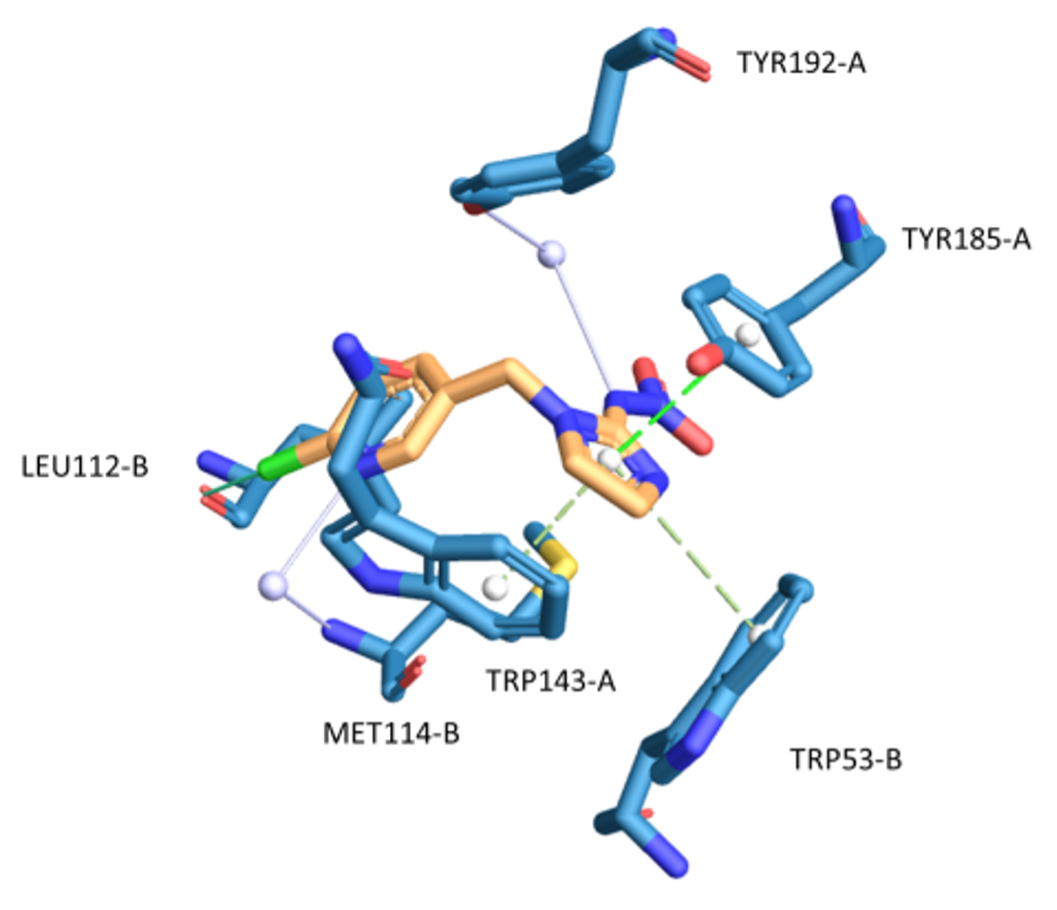  **B** |
| --- | --- |

**S2 Fig.** The 3D structures of the (A) position of IMI between chain A and chain B (B) interactions between IMI and the chain A and B residues.
