# Supplementary material for: In-silico selection of peptides for the recognition of imidacloprid
Source: PLoS One. 2023 Dec 12;18(12):e0295619. doi: 10.1371/journal.pone.0295619 (PMC10715655; doi:10.1371/journal.pone.0295619)
Supplement: S3 Fig — A) The 3D-structure of RNR12 peptide B) The interactions between IMI and RNR12 ligand from molecular docking calculations. (DOCX) [file pone.0295619.s003.docx]

| **A** | **B** |
| --- | --- |
| 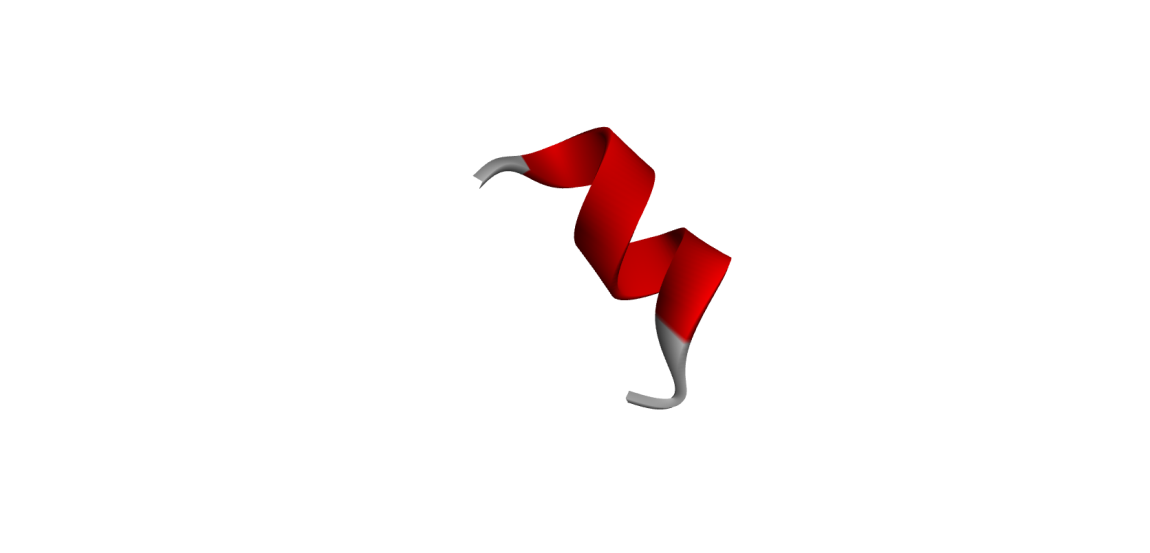 | **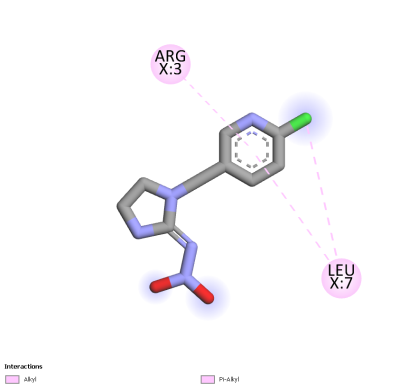** |

**S3 Fig.** A) The 3D-structure of RNR12 peptide B) The interactions between IMI and RNR12 ligand from molecular docking calculations.
