# Supplementary material for: In-silico selection of peptides for the recognition of imidacloprid
Source: PLoS One. 2023 Dec 12;18(12):e0295619. doi: 10.1371/journal.pone.0295619 (PMC10715655; doi:10.1371/journal.pone.0295619)
Supplement: S4 Fig — A) The number of contacts for the peptides vs time B) The minimum number of contacts for each peptide. (DOCX) [file pone.0295619.s004.docx]

| **A**  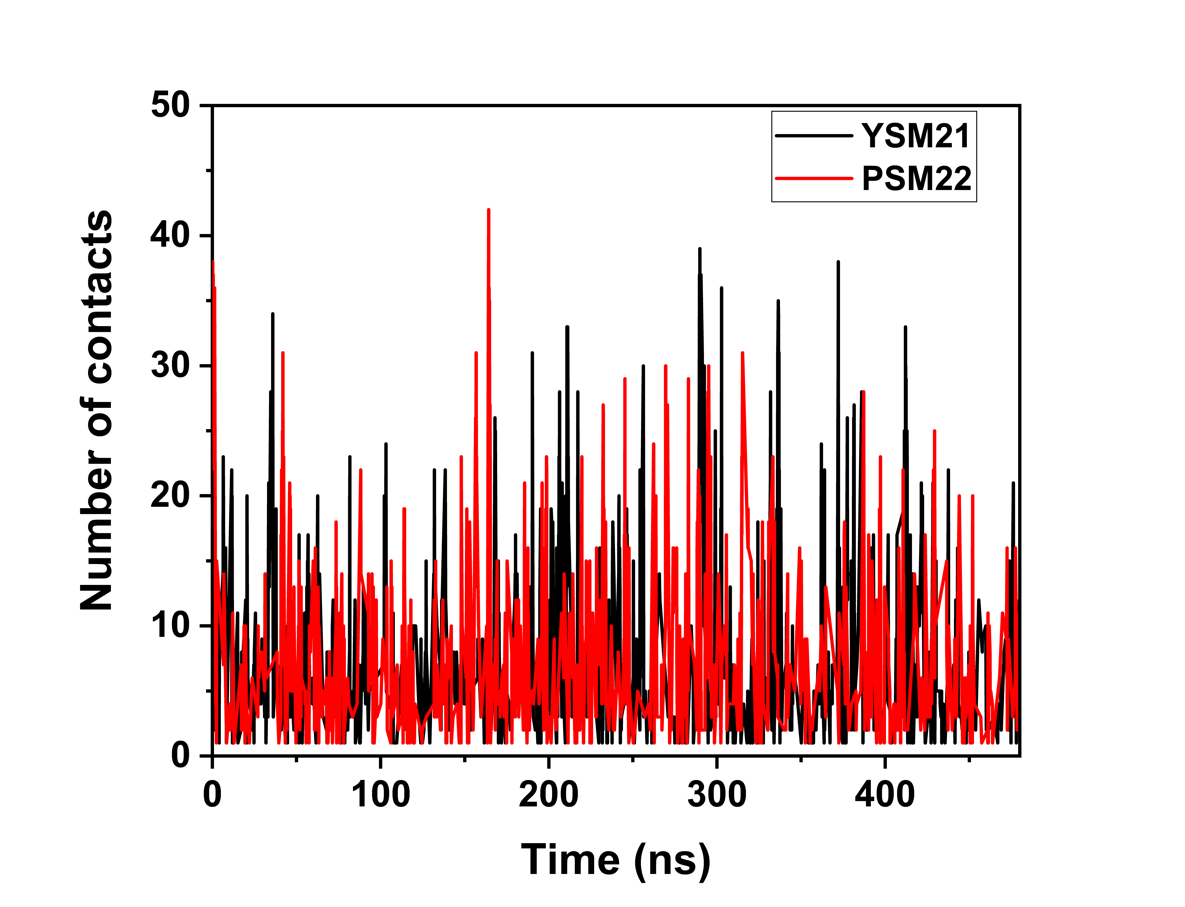 | 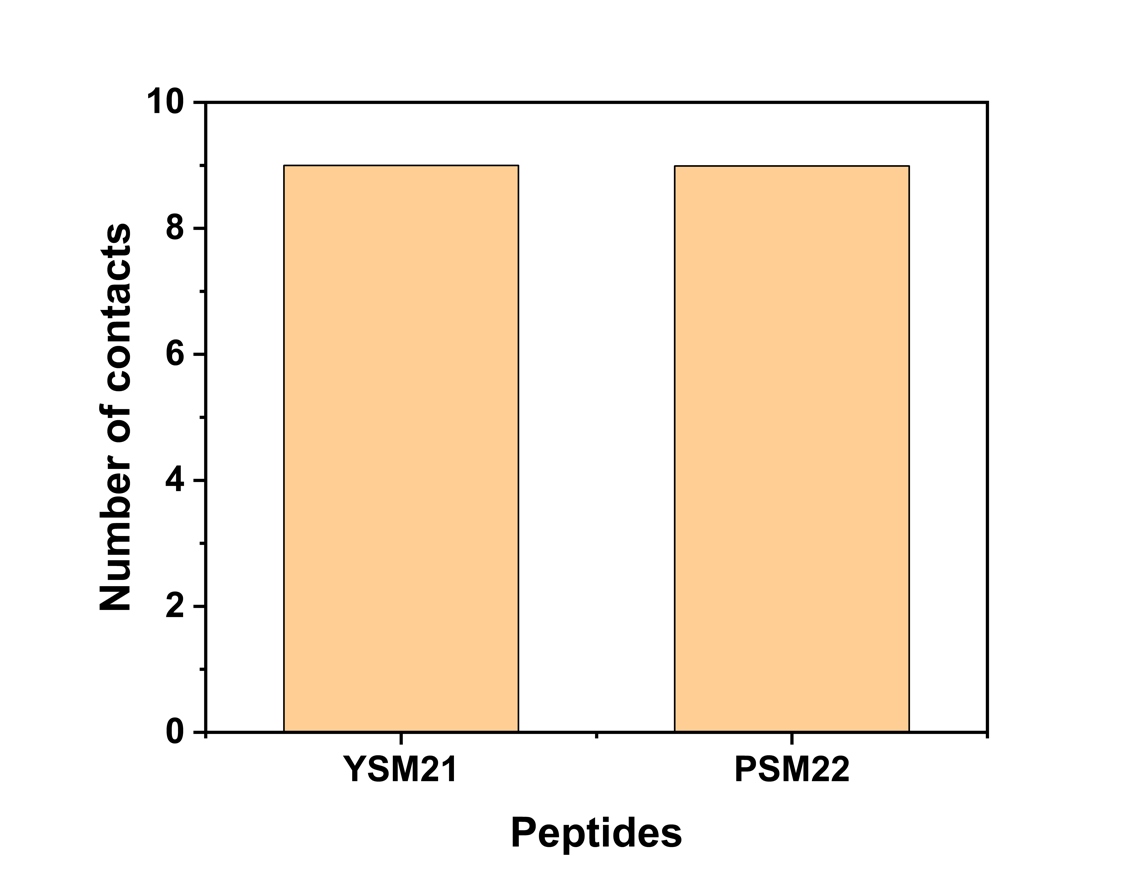  **B** |
| --- | --- |

S4 Fig. A) The number of contacts for YSM21 and PSM22 vs time B) The minimum number of contacts for each peptide.
